# Paradoxical effects of ^137^Cs irradiation on pharmacological stimulation of reactive oxygen species in hippocampal slices from apoE2 and apoE4 mice

**DOI:** 10.18632/oncotarget.20603

**Published:** 2017-09-01

**Authors:** Laura E. Villasana, Tunde Akinyeke, Sydney Weber, Jacob Raber

**Affiliations:** ^1^ Department of Behavioral Neuroscience, ONPRC, Oregon Health and Science University, Portland, OR 97239, USA; ^2^ Departments of Neurology and Radiation Medicine, Division of Neuroscience, ONPRC, Oregon Health and Science University, Portland, OR 97239, USA

**Keywords:** cognition, superoxide, DHE, hippocampus, NADPH

## Abstract

In humans, apoE, which plays a role in repair, is expressed in three isoforms: E2, E3, and E4. E4 is a risk factor for age-related cognitive decline (ACD) and Alzheimer's disease (AD), particularly in women. In contrast, E2 is a protective factor for ACD and AD. E2 and E4 might also differ in their response to cranial ^137^Cs irradiation, a form of radiation typically used in a clinical setting for the treatment of cancer. This might be mediated by reactive oxygen species (ROS) in an-apoE isoform-dependent fashion. E2 and E4 female mice received sham-irradiation or cranial irradiation at 8 weeks of age and a standard mouse chow or a diet supplemented with the antioxidant alpha-lipoic acid (ALA) starting at 6 weeks of age. Behavioral and cognitive performance of the mice were assessed 12 weeks later. Subsequently, the generation of ROS in hippocampal slices was analyzed. Compared to sham-irradiated E4 mice, irradiated E4 mice showed enhanced spatial memory in the water maze. This was associated with increased hippocampal PMA-induction of ROS. Similar effects were not seen in E2 mice. Irradiation increased endogenous hippocampal ROS levels in E2 mice while decreasing those in E4 mice. NADPH activity and MnSOD levels were higher in sham-irradiated E2 than E4 mice. Irradiation increased NADPH activity and MnSOD levels in hemi brains of E4 mice but not in those of E2 mice. ALA did not affect behavioral and cognitive performance or hippocampal formation of ROS in either genotype. Thus, apoE isoforms modulate the radiation response.

## INTRODUCTION

Cancer patients are exposed to ^137^Cs radiotherapy [1, 2]. While whole brain irradiation (WBI) can be a life-saving treatment for brain metastasis, it is associated with detrimental CNS side effects [1, 3]. WBI can lead to progressive and long-term deficits in cognition, including deficits in attention, speed of information processing, executive function, and learning and memory [4].

The temporal lobe, which includes the hippocampus, is sensitive to cognitive changes following irradiation. Following ^137^Cs irradiation in rodent models, impairments in performance on hippocampus-dependent cognitive tests, such as contextual fear conditioning and spatial learning and memory in tests requiring navigation, such as the water maze and Barnes maze, have been reported [5]. Other studies have reported enhanced cognitive performance following irradiation [6, 7]. Hippocampus-dependent tests differ in sensitivity to detect cognitive impairments following a given radiation exposure [8] and irradiation might differentially affect distinct types of hippocampal-dependent memories.

Apolipoprotein E (apoE) is involved in cholesterol and lipid metabolism [9]. Of the three human apoE isoforms (E2, E3, and E4), E4 is a risk factor for age-related cognitive decline (ACD) and Alzheimer's disease (AD), particularly in women [10, 11]. In contrast, E2 is relative protective with regard to AD risk but is a risk factor for more severe post-traumatic stress disorder symptoms [12]. Therefore, it is important to compare the radiation response in E2 and E4 mice. Female wild-type [13] and human apoE [14] mice seem more susceptible to cognitive impairments following space radiation (^56^Fe irradiation; 3 Gy, 600 MeV) than male mice.

Radiation-induced reactive oxygen species (ROS) might mediate the effects of radiation on the brain. The brain is vulnerable to ROS damage, as it has a high oxygen consumption but relatively low levels of antioxidants. Therefore, supplementation of antioxidants might antagonize the effects of irradiation on the brain. The antioxidant alpha-lipoic acid (ALA), naturally present in cells and found in a variety of food products and in FDA-approved over the counter supplements [15], scavenges ROS and chelates redox active transitional metals, thereby inhibiting production of other ROS such as hydrogen peroxide and the hydroxyl radical [16]. ALA also restores age-related reductions in the antioxidant glutathione [17], participates in the recycling of vitamin C and vitamin E, and increases glucose uptake in insulin-resistant neurons [18]. Protective properties of ALA have been shown in the context of a variety of challenges, including irradiation [19]. However, ROS is also required for learning and memory. Therefore, the direction of changes in ROS and cognition is complex. Acute increases in ROS seem beneficial for learning and memory [20]. However, a prolonged increase in ROS can lead to injury through oxidation of cellular components such as lipids, proteins and DNA [21].

Dihydroethidium is a fluorescent dye that is oxidized by superoxide into the stable dihydroxy ethidium (DHE). Based on its ability to detect intracellular and extracellular superoxide, DHE is used for *in vitro* and *in vivo* analysis of superoxide [22], including hippocampal slices of mice [23] and rats [24]. In the current study, we compared behavioral and cognitive performance of sham-irradiated and ^137^Cs-irradiated E2 and E4 female mice and analyzed whether the behavioral and cognitive changes are associated with changes in hippocampal superoxide levels and hemi brain NADPH activity and Manganese Superoxide Dismutase (MnSOD) levels.

## RESULTS

### General health and body weights

**Figure 1:**
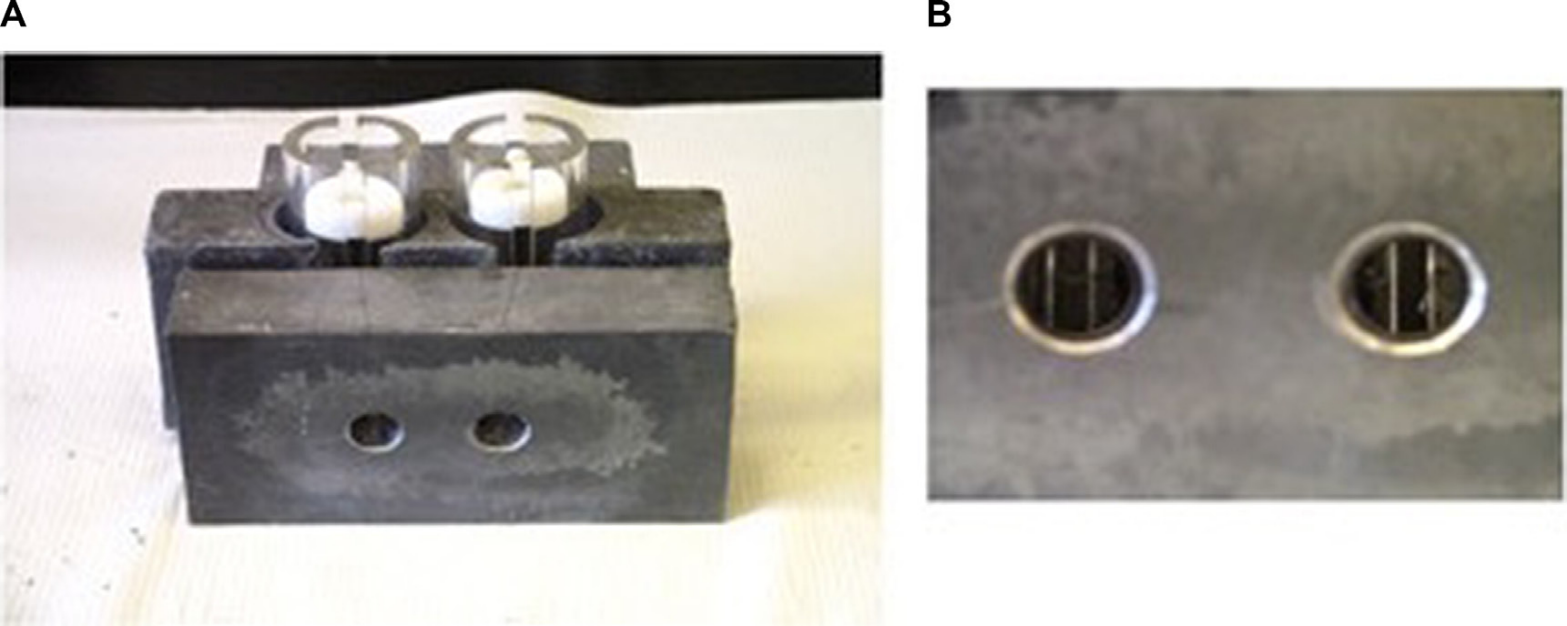
Lead shielding blocks used during ^137^Cs irradiation Following *i.p.* anesthesia, the mice were positioned inside plexiglass tubes containing several ventilation holes. The tubes were placed in a lead block (**A**). Only the back of the heads of mice was exposed through a small opening in the lead block (**B**) to prevent irradiation of non-targeted areas.

Mice were cranially irradiated with ^137^Cs as illustrated in Figure 1. There were no signs of illness or sick-like behaviors as a result of irradiation or diet in either genotype. The body weights of the mice were recorded just prior to irradiation at 8 weeks of age, which was 2 weeks after ALA supplementation, and again at 20 weeks, immediately before the DHE experiments. There were no effects of irradiation or diet in either genotype (data not shown).

### Activity and anxiety measures

**Figure 2:**
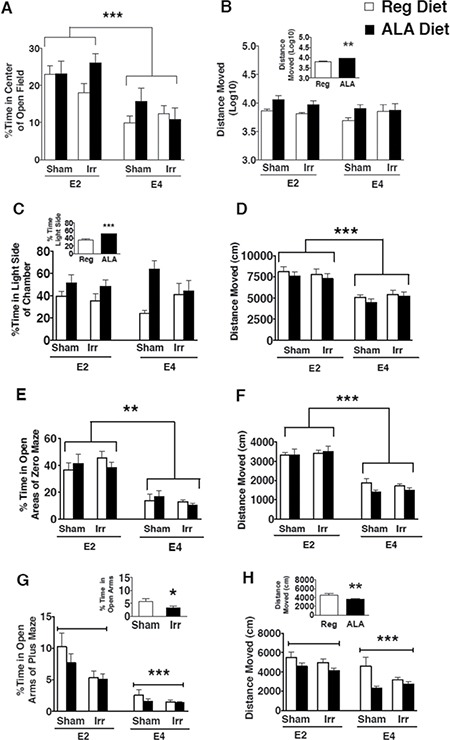
Measures of anxiety and locomotor behavior of sham-irradiated and ^137^Cs-irradiated E2 and E4 mice receiving a regular or an ALA-supplemented diet (**A**) E4 mice spent less time in the center of the open field than E2 mice. (**B**) ALA-supplemented mice moved more than mice fed a regular diet (inset). Group averages for distanced moved were transformed in order to normalize the data. (**C**) ALA-supplemented mice spent more time in the light area of the light-dark enclosure (inset). (**D**) E4 mice moved less than E2 mice in the light-dark test. (**E**) Compared to E2 mice, E4 mice spent less time in the open areas of the elevated zero maze. (**F**) Compared to E2 mice, E4 mice moved less in the elevated zero maze. (**G**) Compared to E2 mice, E4 mice spent less time in the open arms of the elevated plus maze. In addition, irradiated mice spent less time in the open arms than sham-irradiated mice (inset). (**H**) Compared to E2 mice, E4 mice and moved less in the elevated plus maze. In addition, ALA-supplemented mice moved less than mice on a regular diet (inset). **p < 0.01; **p < 0.01; ***p < 0.001; *n* = 7–9 mice per genotype/irradiation treatment/diet.

In the open field test, there was a genotype effect for the percent time spent in the center (*F*_1, 55_ = 28.82, *p* < 0.001). E4 mice spent less time in the center of the open field than E2 mice (Figure 2A). There was a trend towards a genotype × irradiation × diet interaction for the percent time spent in the center of the open field but this did not reach significance (*F*_1,55_ = 3.84, *p* = 0.06). There was an effect of diet on activity levels in the open field (*F*_1, 55_ = 7.14, *p* < 0.01). ALA supplemented mice moved more than regular diet fed mice (Figure 2B, inset).

In the light-dark test, there was an effect of diet on the percent time spent in the light side of the chamber, which is the more anxiety-provoking area (*F*_1, 58_ = 11.52, *p* < 0.001). ALA supplemented mice spent more time in the light area of the enclosure than regular diet fed mice (Figure 2C, inset). There was also an effect of genotype on activity levels in the enclosure (*F*_1, 58_ = 46.76, *p* < 0.001). E4 mice moved less than E2 mice (Figure 2D).

In the elevated zero maze, there was an effect of genotype on the percent time spent in the open areas (*F*_1, 54_ = 62.79, *p* < 0.01). Consistent with the open field data, E4 mice spent less time in the open areas of the elevated zero maze than E2 mice (Figure 2E). There was also an effect of genotype on activity levels in the elevated zero maze (*F*_1, 54_ = 137.46, *p* < 0.001). Consistent with the light-dark data, E4 mice moved less than E2 mice in the elevated zero maze (Figure 2F).

In the elevated plus maze, there was an effect of genotype on the percent time spent in the open arms of the elevated plus maze (*F*_1, 57_ = 40.38, *p* < 0.001). Consistent with the open field and elevated zero maze data, E4 mice spent less time in the open arms than E2 mice (Figure 2G). There was also an effect of irradiation on the percent time spent in the open arms (*F*_1, 57_ = 6.8, *p* < 0.05). Irradiated mice spent less time in the open arms of the elevated plus maze than sham-irradiated mice (Figure 2G, inset). In addition, there was an effect of genotype on activity levels (*F*_1, 57_ = 22.23, *p* < 0.001). Consistent with the light-dark and elevated zero maze data, E4 mice moved less than E2 mice (Figure 2H). Finally, there was an effect of diet on activity levels in the elevated plus maze (*F*_1, 57_ = 10.83, *p* < 0.01). ALA-supplemented mice moved less than regular diet fed mice (Figure 2H, inset).

### Spatial learning and memory in the water maze

**Figure 3:**
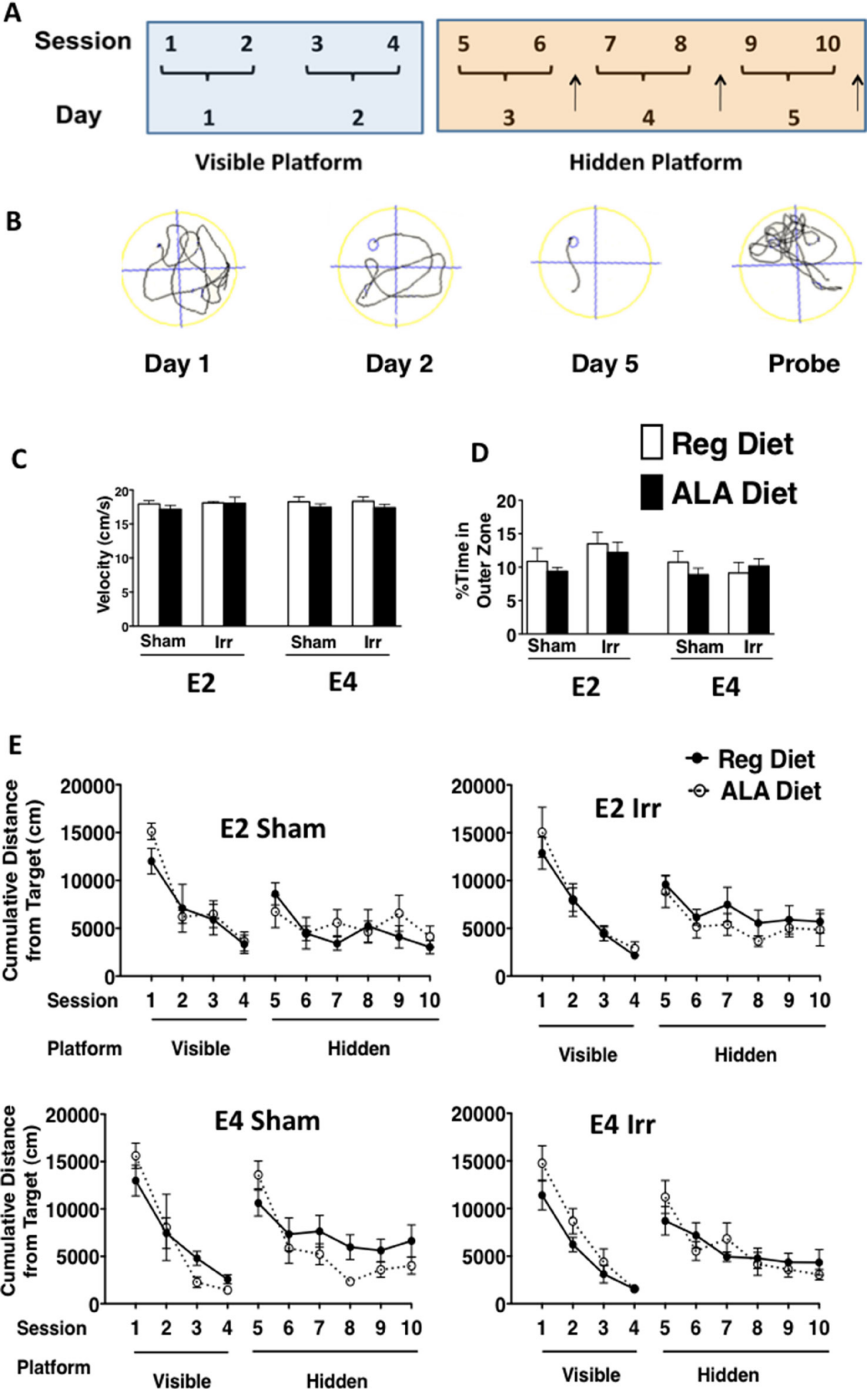
Spatial learning and memory in the water maze (**A**) Illustration of the water maze paradigm. Mice were trained to locate a visible platform, and subsequently were trained to locate a hidden platform. Probe trials (↑) were conducted 1 hour after the last training session of the day, as indicated. (**B**) An example of improved performance (reduced cumulative distance to the target) across training days is shown. The last track in panel B illustrates a target bias, as the majority of the search is close to the target (upper left corner of the pool). (**C**) Swim velocity of sham-irradiated and ^137^Cs-irradiated E2 and E4 mice on a regular or an ALA-supplemented diet during the visible platform training sessions of the water maze. There were no group differences in swim speed. (**D**) Thigmotaxis behavior of the mice. There were no group differences in thigmotaxis. (**E**) Water maze learning curves of sham-irradiated and ^137^Cs-irradiated E2 and E4 mice on a regular or an ALA-supplemented diet. E4 mice showed a greater cumulative distance to the platform than E2 mice. *n* = 7–9 mice per genotype/irradiation treatment/diet.

A diagram of the water maze paradigm and some representative tracks of individual trials are illustrated in Figure 3A, 3B. There were no effects of genotype, irradiation condition, or diet on swim speeds (Figure 3C) or on the percent time spent swimming in the perimeter of the pool, which was analyzed to assess potential group differences in thigmotaxis (Figure 3D). There were no effects of genotype, irradiation treatment, or diet on cumulative distance to the platform location during visible platform training (Figure 3E). There was an effect of genotype on cumulative distance to the platform location during hidden platform training (*F*_3.6, 183.75_ = 2.57, *p* < 0.05). E4 mice showed a greater cumulative distance to the platform than E2 mice, indicating that they swam farther away from the platform location (Figure 3E).

**Figure 4:**
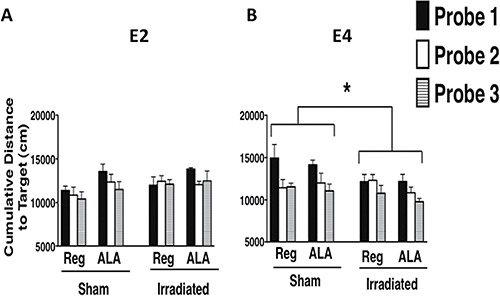
Spatial memory retention of E2 (**A**) and E4 (**B**) mice in the water maze probe trials. ^137^Cs-irradiation-induced enhancement in spatial memory retention of E4 female mice in the water maze probe trials. Across the average of the probe trials, irradiated E4 mice performed better than their sham-irradiated counterparts and better than irradiated E2 mice. *p < 0.05. *n* = 7–9 mice per genotype/irradiation treatment/diet.

Next, spatial memory retention was assessed in the water maze probe trials (no platform). There was a genotype x irradiation condition interaction for average performance across the water maze probe trials (*F*_1, 51_ = 7.89, *p* < 0.01, Figure 4). When the data were split by genotype, the effect of irradiation was significant in E4 mice (*F*_1, 28_ = 6.24, *p* < 0.05). Irradiated E4 mice showed greater spatial memory retention than sham-irradiated E4 mice; irradiated E4 mice showed a lower cumulative distance to the target compared to sham-irradiated E4 mice. Although irradiated E2 mice appeared to show a greater cumulative distance to the target than their sham-irradiated counterparts, particularly on the 2nd and 3rd probe trials, there was no significant effect of irradiation (*p* = 0.18, Figure 4). When the data were split by irradiation condition, there was an effect of genotype (*F*_1, 28_= 5.16, *p* < 0.05). Irradiated E4 mice showed a lower cumulative distance to the target than irradiated E2 mice (Figure 4). There was no genotype difference in sham-irradiated mice (*p* = 0.14). There was also a genotype x diet interaction observed across the average of the probe trials (*F*_1, 51_ = 5.3; *p* < 0.05). When the data were split by genotype, an effect of diet was observed in E2 mice (*F*_1, 27_= 4.21, *p* < 0.05). In E2 mice, ALA-supplemented mice showed a greater cumulative distance to the target compared to genotype-matched regular diet fed mice, indicating that ALA supplementation worsened spatial memory retention in E2 mice (Figure 4A).

### Fear learning and memory

**Figure 5:**
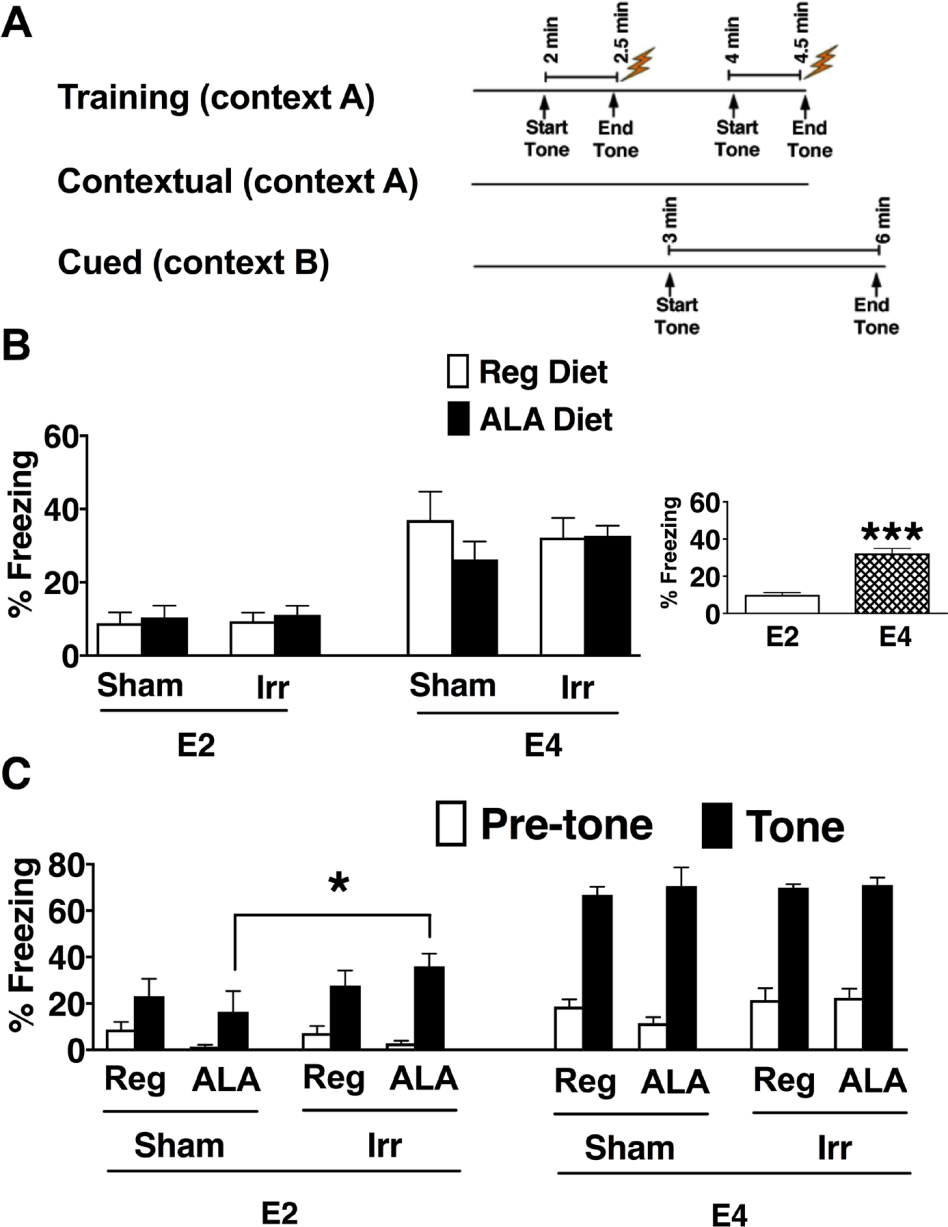
Fear learning and memory (**A**) Illustration of the fear conditioning protocol. The mice received fear conditioning training on day 1. On the second day, context-induced freezing was analyzed when mice were returned to the same environment (context) in which they had received the tone and the two shocks. Freezing behavior was assessed for 3 minutes. One hour later, tone-induced (cued) freezing was analyzed when the mice were placed in a different environment. Freezing behavior was assessed before and after the tone. (**B**) Contextual freezing of sham-irradiated and irradiated E2 and E4 mice on a regular or an ALA-supplemented diet. E4 mice exhibited higher levels of contextual freezing than E2 mice (inset). (**C**) Cued freezing of sham-irradiated and irradiated E2 and E4 mice on a regular or an ALA-supplemented diet. Irradiated E2 mice froze more in response to the tone compared to sham-irradiated E2 mice. *p < 0.05, ***p < 0.001B). *n* = 6–9 mice per genotype/irradiation condition/diet.

Figure 5A illustrates the fear conditioning protocol used in this study. Data for two trials, one training trial and one cued memory trial were lost due to technical problems. Because four chambers were operated by one control box, data for four mice were lost. However, the mice still received the experimental stimuli during these trials (confirmed by the video file). The lost training data were from mice from each of the different four groups. For the cued memory trial, the lost data were from two E2 sham-irradiated mice on the regular diet and from two E2 irradiated mice on the regular diet.

There were no effects of genotype on freezing levels during the baseline period (prior to the first tone) (Kruskal-Wallis test). As the data were not normally distributed, the data for the average and maximum motion index for the first and second shock were transformed (square root). There was an effect of genotype on the response to the shocks (multivariate analysis; *F*_1, 47_ = 19.57, *p* < 0.001, Table 1). E4 mice showed greater motion measures for both shocks in all cases except for the average motion index during shock 1. For that measure, E2 mice showed a greater response than E4 mice (*F*_1, 50_ = 65.95, *p* < 0.001). Other measures in which E4 mice showed greater responses than E2 mice were: average motion, motion index for shock 2 (*F*_1,50_ = 4.23, *p* < 0.05), maximum motion index for shock 1 (*F*_1, 50_ = 8.52, *p* < 0.01), and maximum motion index for shock 2 (*F*_1,50_ = 13.48, *p* < 0.001).

**Table 1:** Baseline measures of freezing and response to a foot shock^1^ of E2 and E4 mice

				Average motion index response		Maximum motion index response	
Genotype	Treatment	Diet	Baseline % freezing	1st Foot shock^***^	2nd Foot shock*	1st Foot shock^**^	2nd Foot shock^***^
E2	Sham	Reg	0	17.4 ± 0.7	28.6 ± 1.4	50.0 ± 2.9	45.9 ± 2.9
E2	Sham	ALA	0	17.0 ± 0.7	29.4 ± 2.3	48.1 ± 3.3	48.1 ± 3.34
E2	Irr	Reg	0	17.2 ± 0.7	28.5 ± 1.8	45.1 ± 2.2	44.0 ± 2.4
E2	Irr	ALA	0	16.9 ± 0.7	30.0 ± 1.9	49.0 ± 1.6	49.1 ± 3.4
E4	Sham	Reg	0.4 ± 0.3	13.7 ± 1.2	32.0 ± 12.0	53.8 ± 3.5	54.5 ± 3.0
E4	Sham	ALA	0.2 ± 0.2	11.5 ± 1.0	29.8 ± 2.2	50.9 ± 3.95	49.6 ± 3.3
E4	Irr	Reg	0	11.4 ± 0.6	32.3 ± 2.9	56.7 ± 4.1	55.8 ± 3.6
E4	Irr	ALA	0.3 ± 0.3	13.1 ± 0.8	30.3 ± 1.5	53.7 ± 3.6	52.2 ± 1.7

^1^Data for the foot shock response were transformed to the square root of the original data in order to normalize the data. Effects of genotye: ***p < 0.001, **p < 0.01 and *p < 0.05.

During the contextual fear memory test, there was an effect of genotype on freezing levels (*F*_1,56_ = 52.74, *p* < 0.001, Figure 5B, inset). E4 mice showed enhanced contextual fear memory and froze more than E2 mice. During the cued fear memory test, there was a tone × genotype × irradiation condition × diet interaction for freezing levels (*F*_1,51_ = 5.2, *p* < 0.05, Figure 5C). When the data were split by genotype and diet, there was an effect of irradiation in E2 ALA-supplemented mice (*F*_1, 3_ = 18.66, *p* < 0.001); irradiated mice showed higher freezing levels than sham-irradiated mice (Figure 5C). This was only significant for freezing levels during the tone (*F*_1,13_ = 5.20, *p* < 0.05) but not for freezing levels during the pre-tone period. When the data were split up by irradiation condition and diet, there was an effect of genotype in every group except for the irradiated mice on the regular diet. In that group, there was an effect of genotype on freezing levels during the tone (*F*_1,12_ = 52.34, *p* < 0.001) but not the pre-tone. Other measures where E4 mice showed greater responses than E2 mice included: sham-regular diet fed mice (*F*_1,12_ = 25.43, *p* < 0.001) for both the pre-tone (*F*_1,12_ = 4.81, *p* < 0.05) and the tone (*F*_1,12_ = 33.28, *p* < 0.001); sham-irradiated ALA-supplemented mice (*F*_1,12_ = 56.48, *p* < 0.001) for both the pre-tone (*F*_1,12_ = 12.5, *p* < 0.01) and the tone (*F*_1,12_ = 57.43, *p* < 0.001); irradiated regular diet fed mice (*F*_1,12_ = 18.09, *p* < 0.001) for the tone only (*F*_1,12_ = 52.34, *p* < 0.001); and irradiated ALA-supplemented mice (*F*_1,15_ = 4.68, *p* < 0.05) for the pre-tone (*F*_1,15_ = 26.7, *p* < 0.001) and the tone (*F*_1,15_ = 28.5, *p* < 0.001).

### DHE oxidation

**Figure 6:**
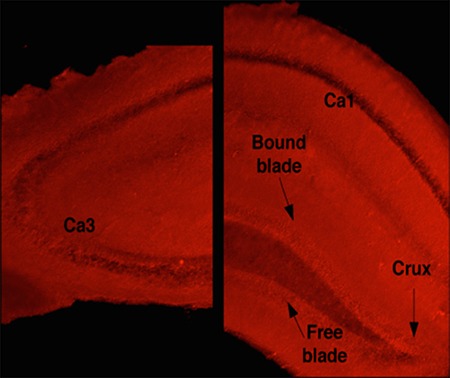
Representative image of a hippocampal slice incubated with DHE Regions of interests included CA1, CA3 and 3 dentate gyrus regions: the crux and the bound and free blades. These regions were traced and analyzed for pixel intensity across 5 time points from 0 to 20 minutes. The autofluorescence (background) of each region for each hippocampal slice was determined and subtracted from the 5 different DHE-oxidation time points assessed.

**Figure 7:**
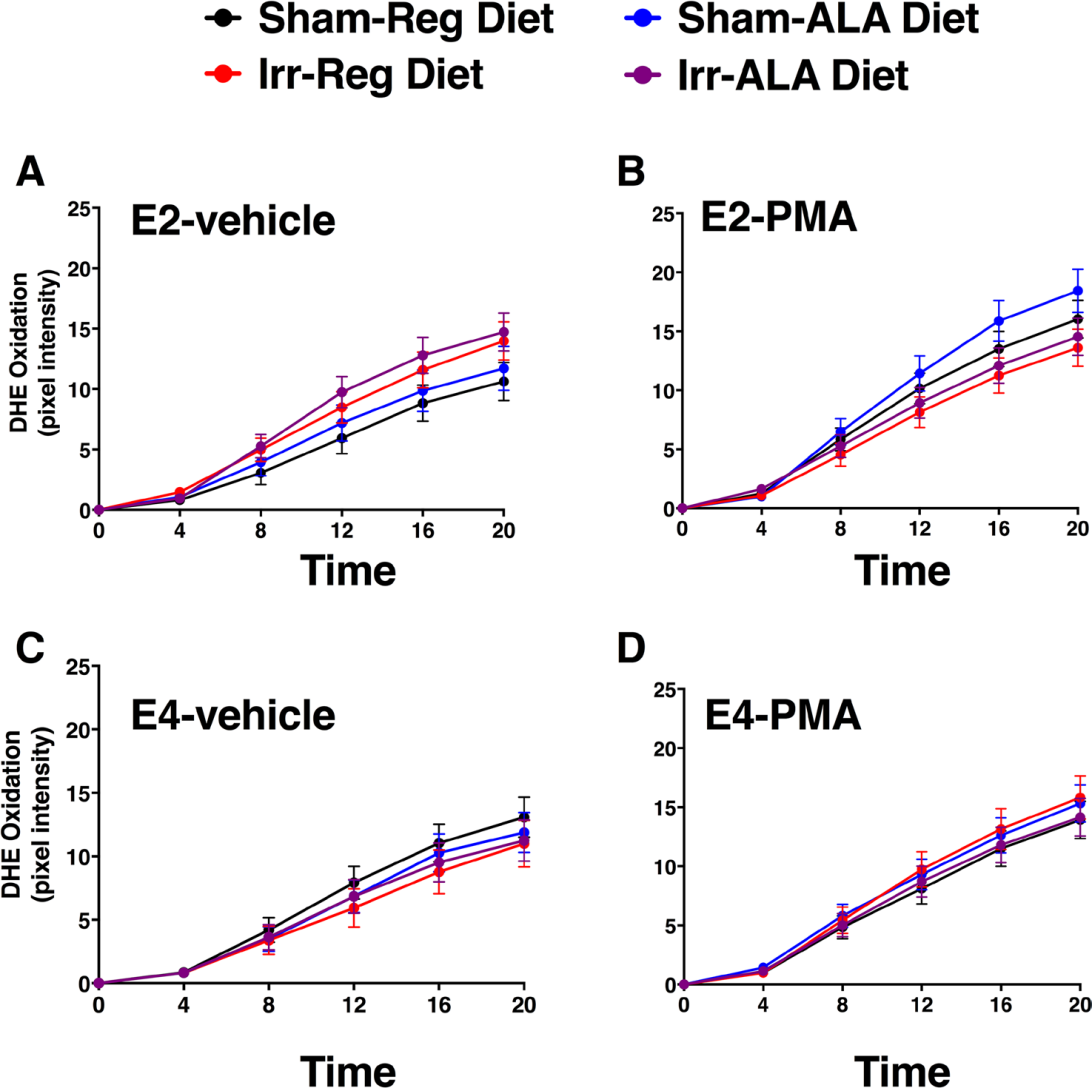
DHE-oxidation of hippocampal slices from all the experimental groups incubated with DHE Graphs show DHE-oxidation of hippocampal slices treated with vehicle (**A**, **C**) or PMA (**B**, **D**). The four different groups within each genotype are presented together for comparison. The ALA diet did not affect levels of ROS. Although diet was not part of the 4-way interaction, note in the top left panel the higher levels of DHE-oxidiation in hippocampal slices from ALA-supplemented irradiated E2 mice (purple) compared to those from sham-irradiated mice on regular diet (black). Data represent the estimated marginal means ± SEM across the average of the hippocampal regions. *n* = 3–4 mice from each each genotype/irradiation condition/diet/drug condition.

**Figure 8:**
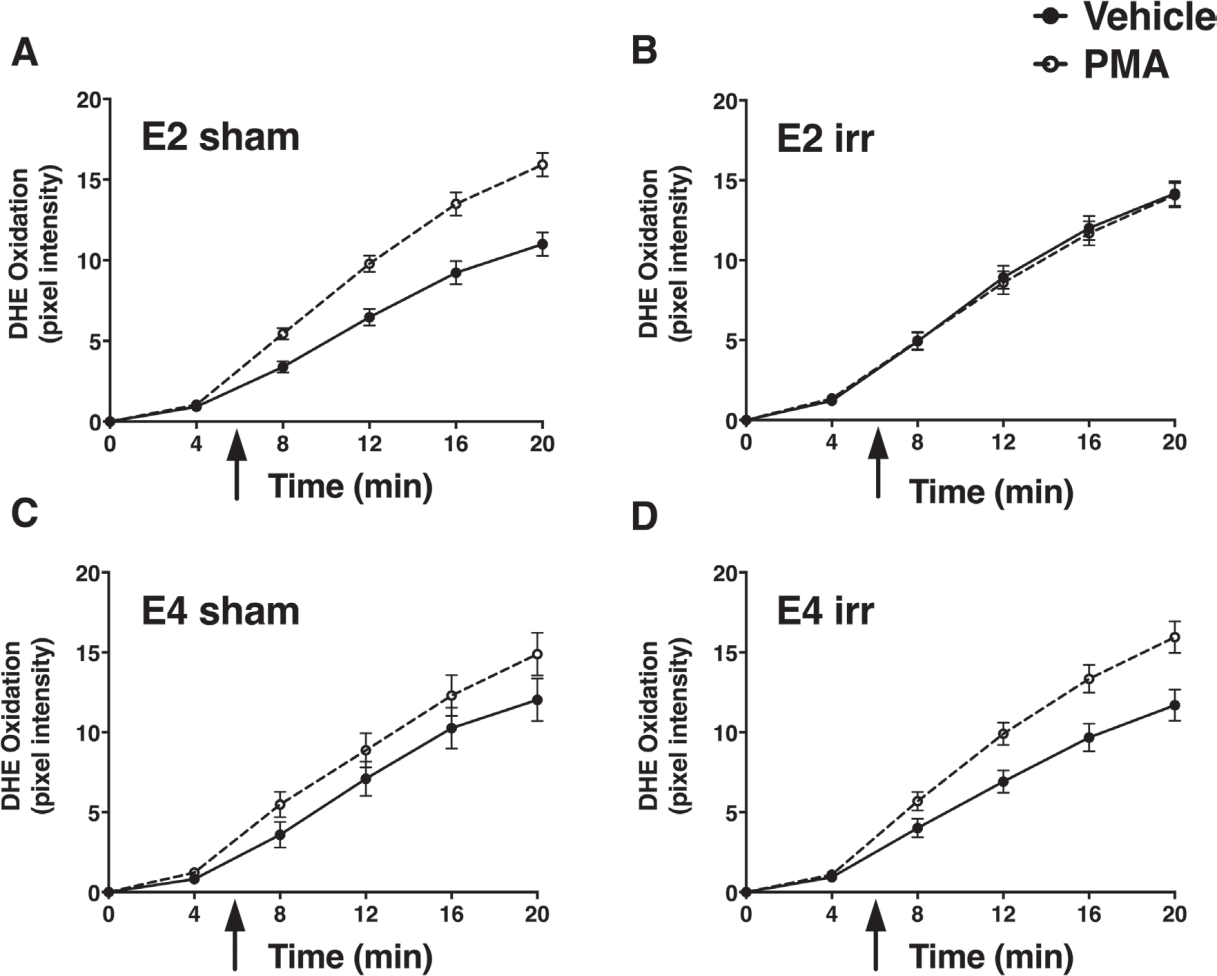
Paradoxical effects of ^137^Cs irradiation on PMA-induced ROS generation in hippocampal slices from E2 and E4 mice Irradiation blunted PMA-induced increases in DHE-oxidation in hippocampal slices from E2 mice (**A**, **B**). In contrast, irradiation enhanced PMA-induced increases in DHE-oxidation in hippocampal slices from E4 mice (**C**, **D**). PMA was added just before the 6 minute time point (arrow). Data represent the adjusted marginal means and ± SEM. *n* = 7–8 mice per genotype/irradiation condition/drug condition.

**Figure 9:**
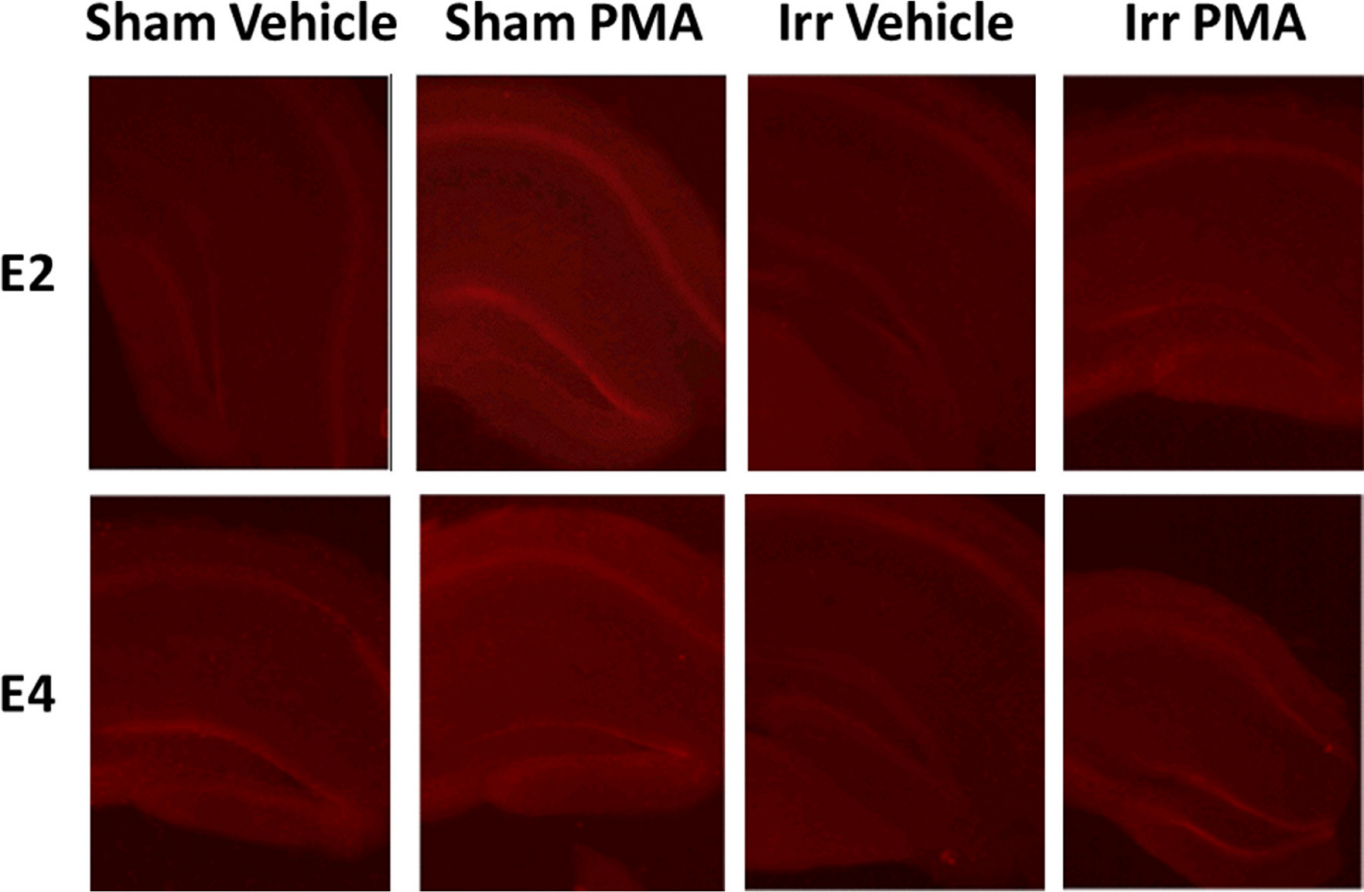
Representative images of PMA-induced DHE oxidation in hippocampal slices from sham-irradiated and irradiated E2 and E4 mice The top images represent slices from E2 mice and the bottom images represent slices from E4 mice.

**Figure 10:**
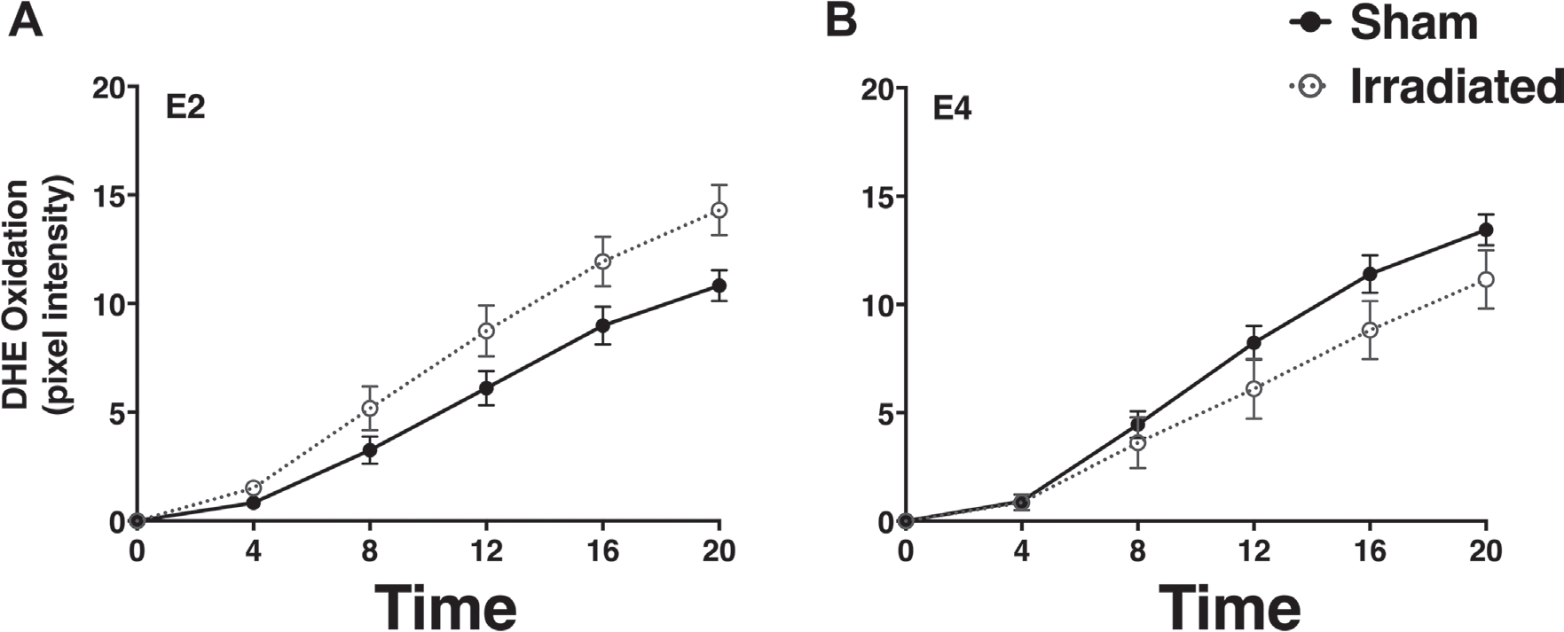
Radiation-induced changes in baseline levels of ROS in vehicle-treated hippocampal slices ^137^Cs-irradiation increased levels of DHE-oxidation in E2 (**A**) but not E4 mice (**B**). Data represent the estimated marginal means and ± SEM. *n* = 7–8 mice per genotype/irradiation condition.

An example of a DHE-treated hippocampal slice with the regions that were examined is illustrated in Figure 6. Analysis of DHE-oxidation revealed a time × genotype × irradiation condition × drug condition (PMA or vehicle) interaction (*F*_1.26,273.83_ = 21.05, *p* < 0.001, Figures 7, 8). As there was no effect of brain region, the data were averaged across the 5 hippocampal areas and used for further analyses. Similarly, as there was no effect of diet, the data for both diets were combined. When the data were split by genotype and radiation treatment to assess the effect of drug treatment, a time × drug interaction was observed in the hippocampal slices from E2 sham-irradiated mice (*F*_1.24,12.44_ = 15.99, *p* < 0.001) and from E4 irradiated mice (*F*_1.34_, 13.44 = 7.09, *p* < 0.05, Figure 8). In both cases, PMA significantly increased DHE-oxidation. Although there was a significant effect of time with genotype, irradiation treatment, and drug, the multivariate analysis for the effect of drug at individual time points did not reach significance. However, there was a significant effect of drug across the time points for the hippocampal slices of sham-irradiated E2 (*F*_1,10_ = 21.21, *p* < 0.001) and irradiated E4 mice (*F*_1,10_ = 8.3, *p* < 0.05, Figure 8). PMA significantly increased ROS generation in slices from sham-irradiated E2 and irradiated E4 mice, but not in slices from irradiated E2 or sham-irradiated E4 mice. Representative DHE hippocampal images for the different experimental groups analyzed in Figure 8 are shown in Figure 9. When the data were then split by genotype and drug to assess the effect of irradiation treatment, a time × irradiation treatment interaction was observed in vehicle-treated hippocampal slices from E2 (*F*_1.53,16.85_ = 7.77, *p* < 0.01) but not from E4 mice. Hippocampal slices from irradiated E2 mice showed higher DHE-oxidation compared to hippocampal slices from sham-irradiated E2 mice (Figure 10).

### NADPH activity and MnSOD levels

**Figure 11:**
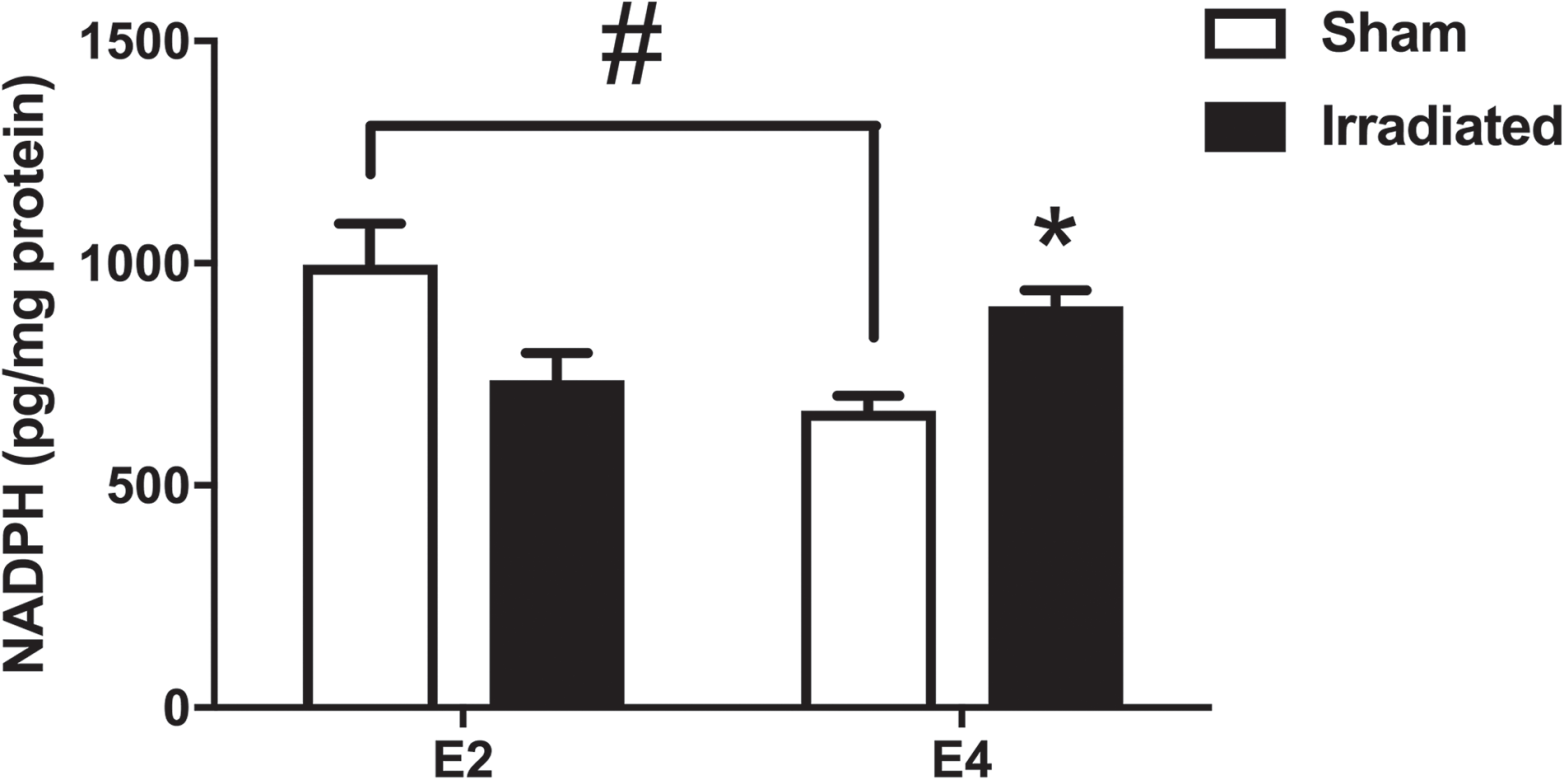
NADPH activity levels in hemi brains of E2 and E4 mice In sham-irradiated mice, NADPH activity levels were higher in E2 than E4 mice. In addition, NADPH activity levels were higher in irradiated E4 mice than sham-irradiated E4 mice. E2 sham-irradiated: *n* = 3 mice; E2 irradiated: *n* = 10 mice; E4 sham-irradiated: *n* = 5 mice; E4 irradiated: *n* = 10 mice. ^#^*p* < 0.05; *p < 0.05 versus sham-irradiated E4 mice.

To assess group differences in sources of ROS, NADPH activity levels in hemi brains of the mice were analyzed. There was a genotype × radiation interaction (*F*(1,24) = 15.28, *p* = 0.0007). In sham-irradiated mice, NADPH activity levels were higher in E2 than E4 mice (*p* = 0.0268 (Tukey's multiple comparisons test), Figure 11). In addition, NADPH activity levels were higher in irradiated E4 mice than sham-irradiated E4 mice (*p* = 0.0365 (Tukey's multiple comparisons test). This was not seen in E2 mice and there seemed a trend towards lower NADPH activity levels in irradiated E2 than sham-irradiated E2 mice (Figure 11).

**Figure 12:**
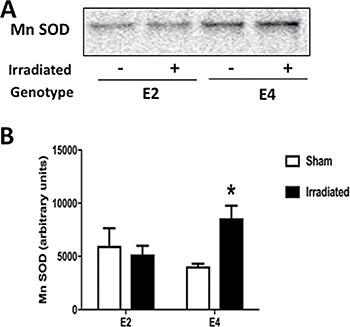
MnSOD levels in hemi brains of E2 and E4 mice (**A**) Representative MnSOD Western blot analysis. (**B**) MnSOD levels were higher in irradiated E4 mice than sham-irradiated E4 mice. E2 sham-irradiated: *n* = 3 mice; E2 irradiated: *n* = 3 mice; E4 sham-irradiated: *n* = 4 mice; E4 irradiated: *n* = 4 mice.

A similar pattern was seen for MnSOD levels. There was a genotype x radiation interaction (*F*(1,10) = 6.422, *p* = 0.0297). MnSOD levels were higher in irradiated E4 mice than sham-irradiated E4 mice (*p* < 0.05, (Tukey's multiple comparisons test), Figure 12). This was not seen in E2 mice and there seemed a trend towards lower MnSOD levels in irradiated E2 than sham-irradiated E2 mice (Figure 12).

## DISCUSSION

This study shows that within the experimental conditions used the effects of ^137^Cs irradiation on spatial memory retention in the water maze are apoE isoform-dependent in female mice and are associated with measures of ROS. Irradiated E4 mice showed greater spatial memory retention than sham-irradiated E4 mice and irradiated E2 mice and this was associated with an increased hippocampal response to PMA-induction of ROS. In E4 mice, hippocampal ROS levels were decreased and hemi brain levels of MnSOD increased following irradiation, while hemi brain levels of NADPH oxidase activity were increased. These data suggest that MnSOD levels might be more important for generation of ROS in the context of ^137^Cs irradiation than those of NADPH oxidase activity. Interestingly, MnSOD levels were also sensitive to effects of ^56^Fe irradiation in E2 mice [23].

Our study makes an essential distinction between higher levels of ROS and the ability to generate ROS upon a pharmacological stimulus. So, it is not simply a matter of having more ROS that is associated with enhanced spatial memory retention, but rather the ability to generate it upon a specific stimulus. The enhanced spatial memory retention of E4 mice following ^137^Cs irradiation is consistent with enhanced hippocampus-dependent contextual fear memory and long-term potentiation in the CA1 region of the hippocampus of C57Bl6/J mice three months following ^28^Si ion (600 MeV, 0.25 Gy) irradiation [25] and cued fear memory of B6D2F1 mice one month following ^16^O irradiation (250 MeV, 0.4 or 0.8 Gy) [26]. Enhanced hippocampus-dependent memory is also seen 2 weeks [27] and 24 hour [28] following post-training X-ray irradiation and might involve reduced GABA-ergic inhibition in parvalbumin-positive cells in the infralimbic cortex [28]. These results are consistent with the notion that performance during a learning and memory task can act as a stimulus for ROS generation [21, 29]. However, it is conceivable that chronic generation of elevated stimulated levels of ROS might cause CNS injury.

In contrast to hippocampal slices from irradiated E4 mice, hippocampal slices from irradiated E2 mice did not show enhanced PMA-induction of ROS. Irradiated E2 mice also did not exhibit radiation-induced enhancements in spatial memory retention in the water maze. These E2 and E4 data support that enhanced hippocampal PMA-induction of ROS following irradiation might mediate enhanced spatial memory retention. A blunted PMA-induction of ROS does not necessarily affect spatial memory retention. A blunted response to PMA was observed in hippocampal slices form irradiated E2 mice, but they did not show changes in spatial memory retention.

The blunted response to the PMA-induction of ROS observed in slices from irradiated E2 mice might be related to changes in baseline levels of ROS. In vehicle-treated hippocampal slices, irradiation increased ROS levels in slices from E2 but not E4 mice. Therefore, it is possible that the blunted PMA response of slices from irradiated E2 might be explained by a general increase in baseline levels of ROS. Another possibility for the blunted response to PMA might relate to potential changes in threshold levels or other factors that can influence induction of ROS. For instance, ROS-induced changes can modulate subsequent responses to ROS induction through adaptive mechanisms [30, 31]. Such effects might involve genotype differences in radiation hormesis, involving adaptive and even beneficial responses to low doses of irradiation and including increases in expression of antioxidants, changes in immune function, and reduction in chromosomal damage through induction of DNA repair mechanisms [32].

The beneficial effects of ^137^Cs irradiation on hippocampus-dependent cognitive function of E4 female mice appear to be limited to spatial memory in the water maze. Irradiation had no effect on associative memory of E4 mice in the contextual fear conditioning test. In contrast to contextual fear conditioning, an effect of irradiation was observed on cued fear conditioning. Specifically, irradiation increased cued fear memory of E2 ALA-supplemented mice, but had no effect on cued fear memory of E4 mice. It is possible that the high levels of freezing exhibited by E4 mice in the cued fear memory test, approximately 80% and twice that seen in the contextual fear memory test, could have masked potential effects of irradiation.

Higher anxiety levels of E4 female mice were previously associated with better performance in the water maze test [35]. However, the enhancing effects of irradiation on spatial memory retention of E4 mice in the water maze are not likely attributed to changes in anxiety-like behaviors. First, there were no group differences in thigmotaxis, a behavior thought be reflective of higher anxiety levels in the water maze task [33]. Second, there was no effect of irradiation during either the visible or hidden platform training sessions. Third, while the effect of irradiation on measures of anxiety in the elevated plus maze was observed in both genotypes, only E4 mice showed an effect of irradiation in water maze. Fourth, irradiation did not affect the performance of E4 female mice in the conditioned fear tasks, which are sensitive to group differences in anxiety levels [34]. Finally, in irradiated E4 mice, the percent time spent in the open arms of the elevated plus maze was not correlated with probe trial performance in the water maze. These data suggest that increased anxiety levels cannot likely explain the enhanced spatial memory retention of irradiated E4 mice in the water maze probe trials. In the anxiety tests, E4 mice showed greater levels of anxiety than E2 mice. Greater anxiety levels of E4 than E2 mice are consistent with our previous studies [12, 35–37].

The effect of irradiation on performance in the elevated plus maze appeared very modest (less than 2% differences compared to sham-irradiated mice) and was not observed in the other anxiety tests. Interestingly, the elevated plus maze also appeared to be more anxiety-provoking than the other tests because mice spent considerably less time in the open arms of the elevated plus maze compared to the center of the open field, light enclosure of the light-dark test, or the open areas of the elevated zero maze. Therefore, it is conceivable that the changes in anxiety levels following irradiation might only be seen in more anxiety-provoking environments.

An effect of ALA was only observed in the light-dark test. ALA-supplemented mice spent more time in the light area of the enclosure compared to mice on regular diet, suggesting that ALA reduced anxiety levels. ALA had opposite effects on total distance moved in the open field test compared to that of the elevated plus maze. In the open field, ALA-supplemented mice moved more, whereas in the elevated plus maze, they moved less. Inconsistent effects of ALA were also previously reported. An ALA-supplemented diet (5% w/w dose for 2 weeks) was associated with increased locomotor behavior in male rats [38] but the same concentration of ALA diet supplementation as used in the current study did not increase locomotor behavior in male mice [39].

ALA did not seem to act as an antioxidant. Analysis of DHE-oxidation showed that hippocampal slices from ALA-supplemented mice were no different than those from mice on a regular diet. This likely explains why ALA did not antagonize the effects of irradiation on spatial memory retention of E4 mice in the water maze. Assuming that the effect of ALA is similar in the amygdala as in the hippocampus, the lack of antioxidant effect of ALA could also explain why ALA did not antagonize the effect of irradiation on cued fear memory of E2 mice. However, ALA had an effect on spatial memory retention of E2 mice in the water maze. Although there were no interactions between diet and genotype for the levels of DHE-oxidation, it appeared that hippocampal slices from irradiated ALA-supplemented E2 mice showed greater DHE-oxidation levels than those from E2 mice on a regular diet. Slices from sham-irradiated E2 mice showed a similar pattern, but to a lesser extent. If ALA acted as a pro-oxidant, it could explain the ALA-induced deficits in spatial memory retention of E2 mice. We based our dose and method of administration on a study that showed reductions in NOS when given to wild-type males at 3-months of age for a duration of 6-months [39]. However, although ALA was associated with a reduction in nitric oxide synthase (NOS), it also resulted in deficits in place recall familiarity, assessed as a reduction in locomotor activity in response to a previously exposed environment. In another study using a similar concentration of ALA in the diet of Tg2576 mice, a mouse model of Alzheimer's disease, ALA reduced memory deficits in contextual fear memory in Tg2576 mice compared to Tg2576 mice that were not given ALA but did not reduce markers of oxidative stress or plaque pathology or affect cognitive performance in wild-type mice [40]. Thus, the effects of ALA-supplementation on measures related to ROS, oxidative damage or on cognitive function are unclear. However, an important difference between the current study and the aforementioned studies is the age at which mice were placed on an ALA-supplemented diet. In our study, mice were placed on an ALA-supplemented diet at 6-weeks of age, whereas in the other studies mice were at least 3-months of age when they were placed on an ALA-supplemented diet. Other potential factors influencing these divergent findings could include the different ROS measures assessed (*e.g.* NOS versus superoxide/hydrogen peroxide), sex, and the genotype of the mice.

In conclusion, this study suggests that radiation-induced increases in ROS can improve hippocampus-dependent spatial memory of individuals with higher background levels of ROS. Because the role of ROS on learning and memory appears to be age-dependent [41], future efforts are warranted to determine the effects of ^137^Cs irradiation on cognitive function and hippocampal ROS generation of older human apoE mice.

## MATERIALS AND METHODS

### Animals

Human E2 and E4 targeted replacement mice, generated by Dr. Patrick Sullivan [42, 43] and bred in our colony, were used for the present study. Two-month-old E2 (sham-irradiated: *n* = 10 mice; irradiated: *n* = 14 mice) and E4 (sham-irradiated: *n* = 4; irradiated: *n* = 6) female mice bred in our mouse colony at OHSU. All mice received *i.p.* anesthesia, (ketamine (Sigma), 80 mg/kg and xylazine (Sigma), 20 mg/kg), and an ophthalmic solution was placed on the eyes for protection. The mice designated for irradiation were placed in positional cradles to stabilize the head during irradiation. Sham-irradiated mice received the same procedure except that they were not irradiated. Upon recovery from anesthesia, the mice were returned to the animal facility. Behavioral and cognitive testing started 12 weeks after irradiation. Water was provided *ad libitum*. See below for the diet information. All procedures were approved by Institutional Animal Care and Use Committee at OHSU.

### Diets

Starting at 6-weeks of age, the mice were placed either on an ALA-supplemented diet (Rodent Pico Chow 20 ± 0.165% ALA, Animal Specialties Inc, Woodburn, OR) or on a regular chow diet (PicoLab Rodent Diet 20, #5053; PMI Nutrition International, St. Louis, MO). The concentration of ALA used in the current study was based on previous reports showing that this concentration attenuates age-related changes in nitric oxide synthase (NOS) levels in male wild-type mice when given at 3 months of age for a 6-month duration [39, 44]. In the current study, mice remained on their selected diets throughout testing.

### Irradiation

Following *i.p.* anesthesia (ketamine (Sigma), 80 mg/kg and xylazine (Sigma), 20 mg/kg), mice were sham-irradiated or ^137^Cs-irradiated (7–9 per genotype per treatment) receiving a dose of 10 Gy using a Mark 1 Cesium Irradiator (Shepherd and Associates, San Fernando, CA) at a dose rate of 1.3 Gy/minute. The cerebellum, eyes, and body were shielded with lead (Figure 1). Mice were housed singly starting 3 days prior to the first behavioral test.

### Behavioral and cognitive testing

Twelve weeks following sham- or ^137^Cs irradiation, mice were behaviorally and cognitively tested as described below. They were first tested in the open field (OF), light-dark test (LD), elevated zero maze (ZM) and elevated plus maze (PM) test. Subsequently, the mice were tested for hippocampus-dependent spatial learning and memory in the water maze (WM) task. Lastly, they were tested for fear conditioning (FC), including hippocampus-dependent contextual fear memory and hippocampus-independent cued fear memory. Approximately one week after the fear conditioning test, hippocampal levels of ROS in mice were determined by DHE-oxidation and hemi brain NADPH oxidase and MnSOD levels were determined, as described below. The experimenter testing the mice was blinded to the treatment of the mice. All tests with the exception of water maze were conducted in the morning. Water maze test sessions were conducted in the morning and early afternoon (beginning at approximately 8:00 am and 1 pm, respectively).

### Open field

The open field was used to evaluate measures of anxiety and locomotor behavior. Mice were placed in a 40.64cm × 40.64 cm brightly lit (luminescence: 200 lux) open arena equipped with infrared photocells interfaced with a computer (Kinder Scientific, Poway, CA). Active times (single beam breaks within 1 second) and distance moved were recorded for a single 10-minute session. In the open-field, the center zone (20.3 × 20.3 cm) is more anxiety-provoking than the peripheral zone. Therefore, mice that are more anxious in the open field spend less time in the center [45, 46]. Total distance moved was used as measure of exploratory behavior and percent time in the center of the open field as a measure of anxiety.

### Light-dark

The light-dark test was also used to assess anxiety levels. In the light-dark test, mice were placed in the open field enclosure (described above) containing black plastic inserts that covered the sides and the top fifty percent of the open field (Hamilton-Kinder, Poway, CA). A single opening in the wall of the insert adjacent to the open area allowed the mice to enter or exit the more anxiety-provoking light area of the maze (luminescence: 200 lux). Active times and distance moved were recorded for a single 10-minute session. Breaks in the photo beams were used to calculate path length, active times, and rest time in the open and closed compartments of the enclosure. Mice with increased measure of anxiety spend less time in the light side of the enclosure [47].

### Elevated zero maze

The elevated zero maze was also used to assess measures of anxiety and exploratory behavior. The custom built elevated zero maze (Kinder Scientific, Poway, CA) consisted of two enclosed areas with two adjacent open areas. Mice were placed in the closed part of the maze and allowed free access for 10 minutes (luminescence: 200 lux). Mice could spend their time either in the closed or open area of the maze. A video tracking system (Noldus Information Technology, Sterling, VA, set at six samples/second) was used to calculate the time spent in the open areas and distance moved throughout the maze. Mice that are more anxious in the elevated zero maze spend less time in the open areas [48]. Outcome measures included the percent time in the open areas and distance moved.

### Elevated plus maze

The elevated plus maze was the last test used to measure anxiety levels [49]. The elevated plus maze consisted of two open arms and two closed arms equipped with infrared photocells interfaced with a computer (Kinder Scientific, Poway, CA). Time in the open arms and distance moved were recorded for a single 10-minute session (luminescence: 200 lux). Recorded beam breaks were used to calculate path lengths and time spent within each arm of the maze. Mice with increased measures of anxiety in this maze spend less time in the open arms [49]. Measures of interest included the percent time spent in the open arms and distance moved.

### Water maze

The water maze test was used to assess spatial learning and memory [50]. A circular pool (140 cm diameter) was filled with water (22°C ± 2°C). The water was made opaque with white chalk in order to hide the platform. The platform (20 cm wide) was located approximately 1 cm below the water level. On the first two days of water maze testing, the mice were trained to locate a visible platform (containing a visible beacon). There were three trials per session (5-minute inter-trial intervals (ITI)) and two sessions (two hours apart) per day. The platform was moved to a new quadrant for each of the four visible platform sessions. Trials ended when the mouse reached the platform and remained on it for 3 seconds or when 60 seconds elapsed. In trials in which the mice did not find the platform, they were guided to the platform and allowed to remain on it for 3 seconds. Upon removal from the maze, the mice were dried with absorbent towels and returned to their home cages.

After visible platform training, the mice were trained to locate a hidden platform in three trials per session (5-minute ITI) and two sessions (two hours apart) per day. The location of the hidden platform remained constant but the drop location varied for each trial. Mice were allowed to remain on the platform for 3 seconds before they were removed from the pool. Performance measures for visible and hidden platform training included swim speeds and cumulative distance to the platform. Cumulative distance to the platform measures how far the mouse is located from the platform over the duration of the trial. The lower the cumulative distance, the better the performance. Thigmotaxis, defined as the percent time spent in the outer 20 cm perimeter of the pool, was analyzed as an anxiety measure in the water maze.

To assess spatial memory retention, probe trials (platform removed) were conducted exactly 1 hour after the last trial of each day of hidden platform training in order. Cumulative distance to the platform location was used as measure of spatial memory retention. The swimming patterns of the mice were analyzed using the Ethovision video tracking system set at 6 samples/sec.

### Contextual and cued fear conditioning

Conditioned fear was used to assess hippocampus-and amygdala-dependent associative memory. In this task, mice learn to associate the environmental context (fear conditioning chamber) or cue (tone) with a mild foot shock (unconditioned stimulus, US). When mice are re-exposed to the context or the tone (conditioned stimuli, CS), conditioned fear results in freezing behavior, which is characterized by cessation of all movement except for respiration. Contextual fear conditioning is thought to be hippocampus- and amygdala-dependent, whereas cued fear conditioning is amygdala-dependent, but not hippocampal-dependent [51, 52]. On the first day of the fear conditioning test, each mouse was placed in a fear conditioning chamber (Med Associates, Inc, St. Albans, VT) and allowed to explore it for 2 minutes before delivery of a 30 second tone (80 dB), which was immediately followed by a 2 second foot shock (0.35 mA). The pre-tone time, which was the first 2 minutes of the trial, was used to assess baseline movement and freezing levels. Two minutes after the first tone-shock pairing, a second tone-shock pair was delivered. Mice were removed from the testing chambers 10 seconds after the second shock and were returned to their home cages. Chambers were cleaned with 0.5% acetic acid between animals. The next day, each mouse was first placed again in the fear conditioning chamber containing the exact same environmental conditions (context) but without delivery of a tone or foot shock. Freezing was analyzed for 3 minutes. The context of the chambers was changed by adding a smooth floor texture over the grid floor, inserting the shape of a triangle, adding a new scent (hidden vanilla soaked nestlets), and by cleaning the chamber with 70% ethanol rather than acetic acid. For each mouse, one hour after the contextual test, cued fear conditioning was assessed. Mice were placed in the chambers containing the modified context and were allowed to explore for 3 minutes before they were re-exposed to the fear conditioning tone for 3 minutes. Freezing behavior was analyzed for the first and last 3 minutes of the cued fear conditioning test.

Freezing was measured using a motion index, calculated based on a proprietary motion analysis algorithm in the Med Associates Video Freeze Software (Med Associates Inc., Georgia, VT). Briefly, the software analyzes and acquires videos of the trials at a frequency of 30 frames/second. The motion index is based on the sum of pixel changes in a frame compared to those of a reference frame and to those of successive frames. The reference frame is based on a video capture when the mouse is not in the chamber. The motion index threshold used in the current study was 18. Thus, a motion index below 18 pixels was considered freezing.

Analysis of group differences in freezing before delivery of the first tone during fear conditioning training on day 1 were analyzed to measure of baseline freezing. This allowed us to determine whether there were potential pre-conditioning group differences in behaviors such as immobility, which could contribute to freezing scores. Potential group differences in the motion index during the two shocks on day 1 were also analyzed. This allowed us to determine whether there were possible group differences in the sensory response to the shocks. Finally, the percent time freezing during contextual and cued testing on day 2 were analyzed.

### DHE oxidation analysis

DHE oxidation levels were assessed approximately one week after fear conditioning in slices from E2 and E4 mice, as described [53]. Hemi brains were placed in 4°C oxygenated (95% oxygen 5% carbon dioxide) cutting solution (in mM: 110 sucrose, 60 NaCl, 3 KCl, 1.25 NaH_2_PO_4_, 25 NaHCO_3_, 0.5 CaCl_2_, 7 MgCl_2_, 5 glucose). Acute brain coronal sections (150 μm) were generated using a Vibratome (Leica Microsystems, St. Louis, MO) containing an oxygenated bath. Sections from each mouse were collected and kept separate in a 12-well plate bath containing ice cold and oxygenated cutting solution. Once all sections were collected, the bath solution was replaced with 50% cutting and 50% artificial cerebrospinal fluid (ACSF) solution (in mM: 125 NaCl, 2.4 KCl, 1.25 NaH_2_PO_4_, 25 NaHCO_3_, 2 CaCl_2_, 1 MgCl_2_ and 25 glucose). Thirty minutes later, the bath solution was changed to a 100% ACSF solution and the bath temperature was gradually increased to 34°C. Sections were transferred and allowed to equilibrate for one hour in a multi-bath chamber placed on an Olympus spinning disk confocal microscope. The multi-bath chamber was situated on the confocal stage and received 36°C oxygenated ACSF using a gravity fed perfusion system and a multiple in-line heater (Warner Instruments, Hamden, CT). The rate of perfusion was 1ml/minute. A superfusion pump was used to perfuse out solution from the chambers, making this an open perfusion system. Each separate chamber contained representative sections from each group of mice. This allowed all the sections from the different treatment groups and two genotypes to be examined under the exact same experimental conditions. Images of the hippocampus (crux, enclosed blade and free blade of the dentate gyrus; areas CA1 and CA3) were acquired for background reference (Ex λ 488 nm; Em λ > 590 nm) before the addition of DHE (10 μM, Molecular Probes, Eugene, OR). Phorbol-12-Myristate-13-Acetate (PMA) induces generation of superoxide via stimulation of the NADPH-oxidase complex [54]. Because induction of superoxide is important for learning, memory, and synaptic plasticity [20, 21, 55, 56], we used PMA as a functional assay to assess whether irradiation alters the ability to generate superoxide in E2 and E4 mice. PMA (1 μM) or DMSO was added to separate ACSF solution reservoirs approximately 5 min following the addition of DHE. Images were acquired every 2 minutes for up to 20 minutes. The optimal x, y, and z coordinates for each slice and each region were selected and programmed before the DHE experiment began. This allowed us to determine the best focal plane and to label the sections with their corresponding mouse ID number. Images were acquired after addition of DHE using a 4× water objective. As there were slight variations in the focal plane within each slice, several images (3–6) were acquired for optimal image quality. An Olympus spinning disk confocal microscope (Olympus Life Science Solutions, Shinjuku, Tokyo, Japan) and Slidebook 6 Digital Microscopy Software (Intelligent Imaging Innovations Inc., Denver, CO) was used to collect the images and analyze the intensity of oxidized DHE. The experimenter that prepared and analyzed the DHE-oxidation of the slices was blinded to the treatment and the genotype. There was a total of four experiments. Each experiment consisted of 2–3 replicate slices for each mouse from each treatment group and for each drug treatment. The mean temperature was 35.9°C with a range of 34.3 –37.3°C between individual experiments. The mean location of the hippocampal slices from Bregma was ^–^2.0 with a range of ^–^1.58 mm-^–^2.4 mm. This anatomical range was selected because previous data suggested that the dorsal hippocampus is more involved in hippocampus-dependent spatial memory than the ventral hippocampus [57].

### NADPH activity ELISA

NADPH oxidase activity levels were analyzed using a mouse NADPH oxidase ELISA kit (MyBiosource, San Diego, CA), according to the instructions of the company.

### MnSOD Western blot analysis

Hemi brains of individual mice were homogenized in RIPA lysis buffer [0.1% SDS, 0.5% sodium deoxycholate, 1% NP-40, 150 mM NaCl, 50 mM Tris pH 8.0] containing the phosphatase inhibitor sodium vanadate [NaV, 1 mM]. Protein lysates were extracted by centrifugation and protein concentrations were determined using the MicroBCA protein assay (Pierce Biotechnology, Rockford, IL). Equal amounts of protein were separated on sodium dodecyl sulfate polyacrylamide (4–12% SDS-PAGE) gradient gels and transferred to a polyvinylidene difluoride (PVDF) membrane. Membrane blots were blocked in Tris-buffered saline and Tween 20 (TBST) [1 × TBS, 0.1% Tween 20] containing 5% milk. The membranes were then incubated with anti-Mn SOD primary antibody [SPC-117, StressMarq Biosciences, Vicoria BC. Canada; Inc, 1:1000] in TBST for 90 min at room temperature. Membranes were washed in TBST [3 × 10 min] before being incubated in donkey anti-rabbit secondary antibody [1:10000] for one hour at room temperature. Membranes were incubated with enhanced chemiluminescence (ECL) reagent (Pierce Biotechnology, Rockford, IL) before being exposed to CL-XPosure Film (ThermoFisher, Rockford, IL) to detect protein changes.

### Statistical analyses

All statistical tests were conducted using SPSS (IBM SPSS, Armonk, NY) or GraphPad Prism software (GraphPad Software, La Jolla, CA) and were considered significant at *p <* 0.05. All figures were generated using GraphPad Prism software. Where relevant, post-hoc corrections for multiple comparisons were applied. Data are reported as averages ± the standard error of the mean. For all statistical analyses, the data were first assessed for normality and homogeneity of variance to determine whether to use parametric or non-parametric statistical analyses. The data distribution was considered normal at a significance of *p* > 0.01 (Shapiro-Wilk test).

## Abbreviations

ACD: age-related cognitive decline
ACSF: artificial cerebrospinal fluid
AD: Alzheimer's disease
apoE: apolipoprotein E
BSA: bovine serum albumin
C: Celcius
CNS: central nervous system
CO: carbon monoxide
Cs: Cesium
DHE: dihydroxy ethidium
E2: apoE2
E4: apoE4
Fig: figure
ITI: inter-trial interval
mM: millimolar
Mn: Manganese
NADPH: nicotinamide adenine dinucleotide phosphate
OHSU: Oregon Health & Science University
PMA: Phorbol 12-myristate 13-acetate
ROS: reactive oxygen species
sec: seconds
SOD: superoxide dismutase
